# Recurrence rates provide evidence for sex-differential, familial genetic liability for autism spectrum disorders in multiplex families and twins

**DOI:** 10.1186/s13229-015-0004-5

**Published:** 2015-05-13

**Authors:** Donna M Werling, Daniel H Geschwind

**Affiliations:** Semel Institute for Neuroscience and Human Behavior, David Geffen School of Medicine, University of California, Los Angeles, 760 Westwood Plaza, Los Angeles, CA 90095 USA; Neurogenetics Program and Department of Neurology, David Geffen School of Medicine, University of California, Los Angeles, 695 Charles E. Young Dr. South, Los Angeles, CA 90095 USA; Center for Autism Research and Treatment, Semel Institute, David Geffen School of Medicine, University of California, Los Angeles, 760 Westwood Plaza, Los Angeles, CA 90095 USA; Center for Neurobehavioral Genetics, Semel Institute, David Geffen School of Medicine, University of California, Los Angeles, 695 Charles E. Young Dr. South, Los Angeles, CA 90095 USA; Department of Human Genetics, David Geffen School of Medicine, University of California, Los Angeles, 695 Charles E. Young Dr. South, Los Angeles, CA 90095 USA

**Keywords:** Female protective model, Sex differences, Multiplex families, AGRE, Recurrence risk, Interbirth interval

## Abstract

**Background:**

Autism spectrum disorders (ASDs) are more prevalent in males, suggesting a multiple threshold liability model in which females are, on average, protected by sex-differential mechanisms. Under this model, autistic females are predicted to carry a more penetrant risk variant load than males and to share this greater genetic liability with their siblings. However, reported ASD recurrence rates have not demonstrated significantly increased risk to siblings of affected girls. Here, we characterize recurrence patterns in multiplex families from the Autism Genetics Resource Exchange (AGRE) to determine if risk in these families follows a female protective model.

**Methods:**

We assess recurrence rates and quantitative traits in full siblings from 1,120 multiplex nuclear families and concordance rates in 305 twin pairs from AGRE. We consider the first two affected children per family, and one randomly selected autistic twin per pair, as probands. We then compare recurrence rates and phenotypes between males and females and between twin pairs or families with at least one female proband (female-containing (FC)) versus those with only male probands (male-only (MO)).

**Results:**

Among children born after two probands, we observe significantly higher recurrence in males (47.5%) than in females (21.1%; relative risk, RR = 2.25; adjusted *P* = 6.22e−08) and in siblings of female (44.3%) versus siblings of male probands (30.4%; RR = 1.46; adj. *P* = 0.036). This sex-differential recurrence is also robust in dizygotic twin pairs (males = 61.5%, females = 19.1%; RR = 3.23; adj. *P* = 7.66e−09). Additionally, we find a significant negative relationship between interbirth interval and ASD recurrence that is driven by children in MO families.

**Conclusions:**

By classifying families as MO or FC using two probands instead of one, we observe significant recurrence rate differences between families harboring sex-differential familial liability. However, a significant sex difference in risk to children within FC families suggests that female protective mechanisms are still operative in families carrying high genetic risk loads. Furthermore, the male-specific relationship between shorter interbirth intervals and increased ASD risk is consistent with a potentially greater contribution from environmental factors in males versus higher genetic risk in affected females and their families. Understanding the mechanisms driving these sex-differential risk profiles will be useful for treatment development and prevention.

**Electronic supplementary material:**

The online version of this article (doi:10.1186/s13229-015-0004-5) contains supplementary material, which is available to authorized users.

## Background

Autism spectrum disorders are developmental disorders that appear early in life and are defined by impairments in social skills and language abilities, as well as restricted interests and repetitive behaviors [1]. These symptoms present heterogeneously, with some autistic children showing severe intellectual disability and poor basic daily living skills, and others with high intelligence and capacity for independence. Overall, current prevalence estimates for autism spectrum disorder (ASD) are approaching 1.5%, at 1 in 68 children, in the United States [2], an increase that is thought to be attributed to increased awareness among parents, physicians, and teachers that leads to more frequent diagnoses. ASD diagnoses are approximately four times more frequent in males than in females, and the mechanisms responsible for this sex difference are not well understood.

Genetic variation contributes strongly to ASD etiology, as evidenced by high concordance rates between twins [3,4] and high recurrence in siblings [5,6], as compared with risk in the general population. ASDs also often appear sporadically as a result of deleterious *de novo* variants that arise in a parent’s germ line. The identification of these rare, *de novo* copy number and single nucleotide variants (CNV, SNV) in ASD cases from simplex families has proven especially fruitful for risk gene discovery in recent years [7-14]. However, the heritable or familial component of ASD’s genetic risk architecture, although likely to account for more than 50% of genetic risk [15,16], is still poorly understood; family-based genetic linkage and association studies have identified very few replicable risk loci [17-26].

Modeling patterns of inheritance have led to the proposal that families with autistic children fall into two risk classes for ASD: a majority of low-risk families in which *de novo* variants are the primary genetic cause of ASD and a minority of high-risk families in which inherited variants follow a dominant transmission pattern for males, with reduced penetrance in females [27,28]. Sibling recurrence risk estimates from multiplex families and from an infant sibling study fit this model, finding ASD recurrence in close to 50% of later-born male children in these families [6,28]. These studies report far lower risk to later-born daughters from multiplex families (approximately 20%), consistent with the effects of a biological mechanism that protects females from manifesting an ASD phenotype.

This model of relative resilience has been termed as the female protective model, a variation on the multiple threshold liability model for ASD risk [29]. According to this model, genetic liability is distributed in the population, and males and females have different thresholds, or minimum variant loads, at which they present an ASD phenotype [30,31]. Following from the model, one would expect affected females to carry a greater risk variant load than affected males on average, and that this variant load, if inherited as opposed to *de novo*, should be shared among siblings. This phenomenon of higher recurrence risk to family members of the less frequently affected sex is referred to as the ‘Carter effect’ [32]. Recent evidence of higher scores on a quantitative measure of autistic traits in siblings of female probands as compared with siblings of male probands in two large, population-based samples supports this hypothesis [33].

At the genetic level, earlier work also observed trends toward higher rates of deleterious CNVs and SNVs among autistic females [7-9,12-14]. A more recent study found a significantly higher rate of CNV and SNV risk variants in females with ASD and other neurodevelopmental disorders, as well as preferential transmission of these CNVs from mothers [34]. However, most genetic studies of rare variants have focused on collections of sporadic ASD cases, which are assumed to show enrichment for *de novo* mutation events (though it is likely that inherited genetic variants contribute to risk among these families as well). It is not known if the effects of increased variant loads in females and their siblings are also evident among multiplex families, which are assumed be enriched for inherited risk variants, in the population or in research collections such as the Autism Genetics Resource Exchange (AGRE) cohort.

AGRE is a collection of pedigree and phenotypic data and genetic material from families with autistic children [35]. Due to an early focus on recruitment of families with multiple affected children, AGRE families have been widely utilized in genetic studies aiming to identify shared, familial risk variants, including linkage analyses [18,20-22,24-26] and family-based association testing [17,23]. The female protective model predicts that prioritizing families with affected females for variant discovery work may enrich study samples for more deleterious and detectable variants with larger effect sizes than the average familial risk variant load. Additionally, understanding the actions of female protective factors could serve to identify new therapeutic avenues.

We analyzed recurrence and concordance patterns in >1,000 multiplex families and >300 twin pairs from the AGRE cohort to test two primary hypotheses based on the female protective model: first, that males will show higher rates of ASD than females, and second, that risk will be greater for the siblings and co-twins of autistic females than siblings and co-twins of autistic males. We additionally posit several related, secondary hypotheses. One is that sex-specific risk and/or protective factors and familial genetic risk variant loads contribute simultaneously to individuals’ total liability for ASD [31], such that: 1) even within families carrying high, female-penetrant genetic liability, females will show lesser risk than males, and 2) male siblings of female probands will show higher risk than male siblings of exclusively male probands. Finally, if we extend the concept of ASD liability to include environmental risk factors, we hypothesize that siblings of autistic males, who are predicted to carry relatively lesser familial genetic liability than siblings of autistic females, will show a stronger relationship between risk-associated environmental factors and the likelihood of ASD diagnosis. If female-specific factors protect against these environmental exposures (as well as against genetic insults), then one can predict that this association between environment and ASD risk will be especially strong for the male siblings of autistic females.

## Methods

### Subjects

AGRE is a collection of phenotypic and genetic data from families with autistic children that was established in 1997 [35,36]. Initially founded as a multiplex cohort, AGRE currently also includes simplex families, though it remains a valuable source of multiplex ASD families for study. All subjects in AGRE provided written informed consent or assent with parental agreement for behavioral evaluation, blood sample collection, and the transfer of collected data to the AGRE program. This study was approved by the Western Institutional Review Board (AGRE), the Institutional Review Board at Washington University (subject recruitment, principal investigator: John Constantino), and by the Medical Institutional Review Board 3 at the University of California, Los Angeles.

Starting from the catalog of all AGRE subjects (database queried on 14 April 2014), which included 12,260 individuals from 2,278 families, we filtered families for inclusion in these analyses (Additional file 1: Figure S1). Extended families were first parsed to nuclear families, and in order to enrich this sample for cases with genetic risk factors as opposed to environmental complications, nuclear families that included a child with pre- or peri-natal insults, or premature birth before 35 weeks, were removed. Families with twin pairs or multiples of unknown zygosity were also excluded.

For this study, we classified as affected all subjects with study diagnoses of autism, ‘broad-spectrum,’ or ‘not quite autism’ based on a clinician’s evaluation of Autism Diagnostic Interview-Revised and Autism Diagnosis Observation Schedule scores. A ‘broad-spectrum’ diagnosis is given to individuals with pervasive developmental disorders of varying severity and includes subjects with conditions formerly termed as pervasive developmental disorder-not otherwise specified (PDD-NOS) and Asperger’s syndrome. A diagnosis of ‘not quite autism’ is given to subjects who meet the autism cutoffs in all symptom domains but who do not meet the age of onset criterion or conversely who meet the age of onset criterion but fall only one point short of autism cutoffs in one or more symptom domains. Families with one or more children with ambiguous diagnoses, in which AGRE clinicians did not evaluate a child but their parent reported a diagnosis from a community professional, were removed from the analysis. All monozygotic (MZ) multiples and dizygotic (DZ) twin pairs from families meeting the above criteria that included at least one affected child were included in concordance analyses.

For analyses of siblings from multiplex families, we applied additional filters. First, since genetic risk variants carried by MZ multiples are non-independent, we selected one individual from MZ sets at random for inclusion. Families with only one remaining affected child were then excluded, as were families in which affected children were half siblings of one another. The birth order of all full sibling children was then assigned by sorting the mother’s or father’s age at time of birth, if known. For the 12 families who lacked parental age information, birth order was assigned by sorting the individual subject identification numbers, which are assigned according to the birth order. The final multiplex sample consisted of 5,328 individuals from 1,120 nuclear families, including 2,404 affected children, 684 unaffected full siblings, and 2,240 parents.

Each multiplex nuclear family was then classified by the sex of the first two affected children born in the family (probands) either as FC with at least one affected female proband or as MO with only affected male probands. While this approach will misclassify some families with later-born autistic daughters as ‘male-only,’ it prevents the artifactual inflation of recurrence rates in FC families (and deflation in MO families) that results from calculating recurrence rates in the same, later-born children that are also considered during family classification. In other words, if the sex of all affected children in a family is considered during family classification, then later-born affected girls will always contribute positively to the FC recurrence rate, while all female children in MO families will be unaffected by definition, thus reducing the apparent recurrence in MO families. Using the sex of the first two affected children born to classify each family therefore allows us to more definitively separate MO from FC families than is possible from a single proband but does not systematically bias the recurrence rates that we observe in children born after the probands.

### Sex ratios

We calculated the ratio of males to females from all affected children in the multiplex family set. Then, since previous studies have shown differences in the relative numbers of affected males and females among high- and low-functioning cases [37,38], we also calculated sex ratios within the subsets of affected children who met the criteria for the strict autism diagnosis, children with lesser diagnoses of broad-spectrum or not quite autism, children with a Vineland Adaptive Behavior Scales (VABS) composite standard score within the top quartile in the sample (score ≥75), and children with a VABS score within the bottom quartile (score ≤50) [39]. We applied the VABS as the main measure of interest here as it is the most completely ascertained phenotypic measure of intellectual ability or general functioning in AGRE, with 1,656 of 2,404 (69%) cases with recorded scores. Though we do utilize the full range of recorded VABS scores for an additional assessment of quantitative phenotypes, here, we simply use the top and bottom quartiles of the VABS scores as a proxy for the most high- and most low-functioning cases within this data set.

### Recurrence risk

To determine if multiplex families from AGRE show evidence of a female protective effect for ASD penetrance, we assessed ASD prevalence in siblings beyond the two affected children required per family to meet criteria for multiplex status. We then tested whether this risk differs by the sex of the evaluated children or by the families’ classification as MO or FC.

In all families with additional children born after their second affected child, we assessed recurrence risk. First, we recorded the affection status of all children born after the second affected child (*N* = 456 children from 341 families); this provided the largest available sample of latter-born children for estimating recurrence rates. Next, we estimated recurrence in three variations of this sample in order to give equal weight to each nuclear family regardless of size and to directly replicate and extend previous analyses of recurrence in the AGRE sample.

In the first variation, we recorded the affection status of only the first child born after the second affected (*N* = 341 families); this method ensures that all families contribute independently to the risk calculation but limits sample size. In the second variation, we replicated the method applied in a 2007 study of recurrence risk in AGRE families [28] by recording the affection status of the third child from families with exactly three children in which the first two children are affected with ASD (*N* = 198 families). This strict approach was applied to control for effects of ‘stoppage,’ or parents’ decisions to curtail their intended family size after having children with ASDs, on recurrence risk estimates. Here, for the third variation, we also extended this strategy to test the last-born child in all families who had only one additional child after their second affected, regardless of the total family size or birth order of the first two affected children. This extension allowed us to include a greater number of families (*N* = 258 families) than that used in the method from Zhao *et al*. [28] while still controlling for potential stoppage effects.

To evaluate the risk across multiplex families without limiting this analysis to later births in families who continued having children and without weighting these estimates by including multiple children from large families, we next calculated what we refer to as ‘familial risk.’ The main purpose of this analysis was to determine whether the patterns we observe in sample variations of latter-born children are also reflected in the larger sample of multiplex families from AGRE. In all families with at least three children, we calculated ‘familial risk’ by running 1,000 trials in which two affected children are masked at random and affection status is evaluated in a third child. Per trial, the sexes of the masked affected are also used to classify the family as FC or MO. Familial risk is taken as the mean risk from these 1,000 randomizations. This method allowed for the inclusion of those families who stopped having children after their second affected, as well as all children in each family regardless of birth order. Since only one child per family was considered in each randomized trial, this approach also ensured that large families would not contribute disproportionately to the risk estimate.

For each of these five estimates of recurrence or familial risk - (A) all subsequent children from all families, (B) the single next-born child from each family, (C) the third-born child from three-child families, (D) the last-born child from families where the second affected child is born second to last, and (E) familial risk from 1,000 random selections of one child per family - we performed one-sided Fisher’s exact tests in JMP (SAS Institute, Inc., Cary, NC, USA) to compare the risk in males to females and in FC to MO families. We also compared the males’ and females’ risk within FC families and males’ risk in FC to males’ risk in MO families. *P* values were adjusted for these 20 tests by Bonferroni correction. Additionally, we applied logistic regression models to test for interaction effects of sex by family type.

### Birth order and interbirth interval

Earlier work has reported increased ASD risk for children born shortly after elder siblings in population samples [40-42]. It is not known how a risk factor such as short interbirth interval (IBI) interacts with genetic risk profile. Additionally, though one might assume that risk for ASD is constant across the births in a family who share a common source of heritable genetic risk variation, it may be that risk for ASD is increased in later-born children due to the additional accumulation of deleterious variants in the germ line with increasing parental age [43], for example. So, we investigated the relationships between birth order and IBI on recurrence risk in our family sample. For birth order, we assessed differences in ASD recurrence rates for children born first versus second after a family’s second affected child. Risk was evaluated separately by subjects’ sex and family type, and two-sided Fisher’s exact tests were applied to identify significant differences in risk between birth order positions. For IBI, we used parents’ age at the time of each child’s birth to calculate the number of months in between siblings’ births. Using logistic regression for affection status by the natural log of IBI in months, we then tested the relationship between IBI and ASD risk separately by sex and family type (MO or FC) in the child born first after the second affected child from the 332 families with complete parental age information. Maternal and paternal ages were considered as covariates. Pregnancy and birth complications were not explicitly considered in this model, since all families with any records of pre- or peri-natal insults or premature birth before 35 weeks were excluded from this and all analyses in this report.

### Quantitative phenotypes

Previous studies have reported an exacerbation of the male bias for ASD among high-functioning individuals and a greater representation of females among cases with intellectual disability [37,38,44,45]. In contrast, the female protective model predicts that males, who lack female protective factors, should be more severely impacted than females by genetic risk loads of comparable magnitude; this differential impact may be detectable as shifts in phenotype severity, measured quantitatively. We tested several quantitative phenotypes related to ASD severity and intellectual ability, including the VABS composite standard score [39] (1,656 recorded scores - 69% of cases), the Peabody Picture Vocabulary Test (PPVT) standard score [46] (1,386 recorded scores = 58% of cases), the Raven’s Progressive Matrices estimated non-verbal intelligence quotient (Raven’s NVIQ) [47] (1,316 recorded scores = 55% of cases), and the Social Responsiveness Scale (SRS) raw total score [48] (1,042 recorded scores = 43% of cases), for sex differences overall and within FC families, and for differences between MO and FC families. Affected subjects missing scores on these measures were more likely to have been ascertained early on in AGRE’s collection than more recently (as approximated by the sequentially assigned ID number for each family; difference in ID index between subjects with missing versus recorded scores for VABS = −487.93, standard error (std. err.) = 46.11, *P* < 1e−04; PPVT diff. = −385.96, std. err. = 44.47, *P* < 1e−04; Raven’s NVIQ diff. = −282.98, std. err. = 44.37, *P* < 1e−04; SRS diff. = −0.563, std. err. = 43.36, *P* = 0.99) and were also more likely to have been born later in their family (difference in birth order between subjects with missing versus recorded scores for VABS = 0.047, std. err. = 0.043, *P* = 0.27; PPVT diff. = 0.24, std. err. = 0.040, *P* < 1e−04; Raven’s NVIQ diff. = 0.24, std. err. = 0.039, *P* < 1e−04; SRS diff. = 0.14, std. err. = 0.038, *P* = 2e−04), than affected subjects with recorded scores.

We used scores as recorded by AGRE, and in cases where a child was evaluated more than once, we used the most recent score for analysis. All scores recorded as ‘untestable’ were set to missing. For the Raven’s NVIQ, some children received scores of ‘ATN’ (above the highest possible NVIQ score normalized by age; *N* = 93) or ‘BTN’ (below the lowest possible NVIQ score normalized by age; *N* = 19). These scores were recoded as 160 and 20, which are above the observed maximum and below the observed minimum NVIQ scores in the remaining subjects. These high and low values match the maximum and minimum scores for the PPVT and VABS standard scores, two metrics that are scaled analogously to standard IQ.

Scores for the VABS, PPVT, and Raven’s NVIQ are positively correlated with one another, with correlation coefficients of 0.447 (VABS with Raven’s NVIQ), 0.545 (PPVT with Raven’s NVIQ), and 0.595 (VABS with PPVT). As higher SRS scores indicate more severe ASD traits, SRS is negatively correlated with the above measures (*r* = −0.291 with Raven’s NVIQ; *r* = −0.395 with PPVT; *r* = −0.573 with VABS). However, each of these instruments measures a different aspect of abilities (adaptive behavior, vocabulary ability, non-verbal intelligence) or symptoms (traits specifically associated with the ASD phenotype), and so, we opted to include all of these tests in our analyses.

Sex and family classification comparisons were assessed by *t*-tests allowing for unequal variances in JMP using the scores from one proband selected at random from each nuclear family. To test for phenotypic differences between FC and MO families that are not potentially confounded by potential sex differences in phenotypic measures, randomly selected male probands were also compared. To test for sex-differential phenotypes within FC families, a paired *t*-test was used to compare scores from one randomly selected affected female and one affected male within each family. *P* values were adjusted for 16 tests by Bonferroni correction.

### Concordance in twin pairs

MZ (111 twin pairs and 1 set of quadruplets) and DZ twins (193 pairs) with at least one affected member from families without perinatal complications or ambiguous diagnoses were evaluated for ASD concordance. MZ multiples were stratified by their sex (female-female (F-F) and male-male (M-M)) and tested for concordance rate differences using the one-sided Fisher’s exact test (F-F > M-M). For DZ twin pairs, we selected one affected twin from each pair as the proband twin, and we compared ASD recurrence rates in the co-twin by the sex of the co-twin and the sex of the proband (analogous to FC versus MO comparison for siblings) using a one-sided Fisher’s exact test (male co-twin > female co-twin and female proband > male proband). We also used a logistic regression model to test for an interaction effect of proband sex by co-twin sex on ASD recurrence rates in this sample of DZ twins.

## Results

### Sex ratios

Within 1,120 nuclear families with two or more full sibling children with diagnoses of ASD, there are 2,404 affected children, including 1,867 affected males and 537 affected females for an overall male-to-female ratio in these families of 3.48 (Table 1). In contrast with reports of even greater male skew among less severely affected cases [37], the sex ratio for children with AGRE diagnoses of broad-spectrum and NQA (‘not quite autism’) was 2.11, compared with 3.71 for children diagnosed with autism. A comparison of sex bias within cases scoring in the top and bottom quartiles from this sample on the VABS, the most completely ascertained measure of general functioning in the sample, showed a similar pattern, with a greater proportion of affected females falling in the high functioning quartile of the scale (M:F = 2.35) than the lower functioning quartile (M:F = 4.05). We note that these unexpected patterns may not accurately reflect trends at the general population level and may instead be a consequence of the multiplex ascertainment scheme for AGRE.

**Table 1:** **Ratio of affected males to females in multiplex families from AGRE**

	**Number of families**	**Number of affected children**	**Number of affected males**	**Number of affected females**	**Male:Female**
All diagnoses^a^	1,120	2,404	1,867	537	3.48
Autism	1,106	2,158	1,700	458	3.71
Spectrum^b^	220	246	167	79	2.11
Top quartile (≥75) VABS^c^	319	445	312	133	2.35
Bottom quartile (≤50) VABS^c^	306	424	340	84	4.05

^a^All diagnoses = autism, broad-spectrum, or not quite autism (NQA) study diagnoses; ^b^Spectrum = broad-spectrum or NQA; ^c^VABS quartiles calculated from composite standard scores for affected children from AGRE multiplex families. VABS, Vineland Adaptive Behavior Scales.

### Recurrence risk

**Figure 1:**
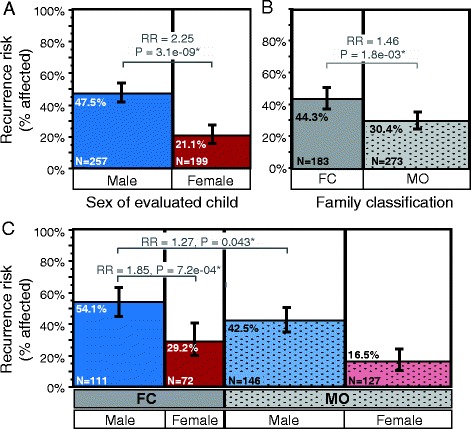
**Recurrence risk for ASD in multiplex families from AGRE by sex and family type.** ASD risk to all children born first after the second affected child in each family (*N* = 456 children from 341 families) is highest for males and in female-containing families. Mosaic plots show the proportion of affected children by **(A)** sex, **(B)** family type as FC (female-containing) or MO (male-only), and by **(C)** sex within each family type. Bar widths are proportional to the number of children or families in each group, which is also noted in the figure. Whiskers note the 95% confidence intervals around each recurrence rate estimate.

The recurrence rate for ASD in the multiplex set of families with at least one child born after the second affected (*N* = 456 children from 341 families) was 36.0% (Additional file 2: Table S1A). The recurrence rate in male children was 47.5% and 21.1% in female children, a significant difference (*P* = 3.11e−09, adj. *P* = 6.22e−08), representing a male to female (M:F) relative risk (RR) of 2.25 (Figure 1A); these sex-differential rates closely match those observed by Zhao and colleagues analyzing a sample of 165 AGRE families [28]. We also observed a difference in risk between FC and MO families, with 44.3% recurrence in FC and 30.4% recurrence in MO families (*P* = 1.78e−03, adj. *P* = 0.036) for a RR of 1.46 for FC families compared with MO (Figure 1B). The sex difference in recurrence risk between males and females within FC families was robust, with RR of 1.85 (*P* = 7.18e−04, adj. *P* = 0.014), and the difference between males from FC and MO families was nominally significant (RR = 1.27, *P* = 0.043, adj. *P* = 0.86; Figure 1C). When only the first child born after the second affected child was included (*N* = 341 children), we find a RR of 2.36 in males compared with females (*P* = 1.21e−08, adj. *P* = 2.42e−07) and 1.27 in FC compared with MO families (*P* = 0.051, adj. *P* = 1; Additional file 2: Table S1B). Sex-differential risk was again apparent within FC families, with RR of 2.07 (*P* = 7.19e−04, adj. *P* = 0.014).

Since parents’ decision to curtail their intended family size after having an autistic child, or stoppage, impacts analyses of family structure, we calculated recurrence risk from 198 families with a specific structure: exactly three full sibling children, with affected first and second children, as was used to estimate recurrence rates in an earlier study of AGRE families [28]. Recurrence risk patterns in this specific set were comparable to those from all families with additional births, with 38.9% risk overall, a RR of 2.39 in males versus females (*P* = 9.57e−06, adj. *P* = 1.91e−04), RR of 2.31 in males versus females from FC families (*P* = 1.86e−03, adj. *P* = 0.037), and a RR of 1.46 in FC versus MO (*P* = 0.025, adj. *P* = 0.49; Additional file 2: Table S1C). When this test is expanded to consider all families who had only one more child after their second affected (*N* = 258 families), recurrence risk again follows the same pattern (M:F RR = 2.41, *P* = 6.87e−07, adj. *P* = 1.37e−05; M:F in FC families RR = 2.06, *P* = 3.59e−03, adj. *P* = 0.072; FC:MO RR = 1.43, *P* = 0.02, adj. *P* = 0.39; Additional file 2: Table S1D). The difference between males from FC versus MO families only reached nominal significance in the set of 198 families with three children (RR = 1.44, *P* = 0.034, adj. *P* = 0.68).

We also calculated familial risk by applying a randomization procedure that permitted inclusion of all 556 families with at least three full sibling children in the analysis. Familial risk in these families was found to be 17.5% (Additional file 2: Table S1E), less than the overall recurrence risk. This is likely a simple consequence of including 221 families with unaffected, earlier-born children; these families do not contribute to any recurrence rate calculations. The absolute familial risk estimates within each sex and family classification were similarly low, at 25.56% for males and 9.79% for females and 22.60% for FC and 14.37% for MO families. Relative risks showed slightly more pronounced differences than for recurrence risk (M:F RR = 2.61, *P* = 6.70e−07, adj. *P* = 1.34e−05; FC:MO RR = 1.57, *P* = 9.72e−03, adj. *P* = 0.19). Logistic regression for affection status in the later-born children from each of the family sets described above, or the non-masked, randomly selected children from familial risk calculations, additionally demonstrated significant main effects of sex and family type on risk for ASD. However, in all family sets tested, the interaction between sex and family type did not reach significance (Additional file 2: Table S2).

### Birth order and interbirth interval

**Figure 2:**
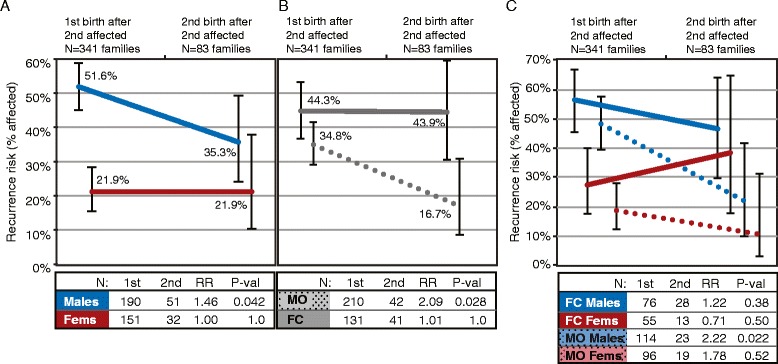
**Patterns of ASD risk to children born after the multiplex criterion are met.** After a family has two affected children, ASD recurrence risk is less for later-born males, particularly those in male-only (MO) families. Line graphs show the proportion of affected children born first and second after a second affected child in all families with at least one subsequent birth by **(A)** sex, **(B)** family type as female-containing (FC) or MO, and **(C)** by sex within each family type. Line plots corresponding to each group are indicated by colors (blue for males, red for females) and solid (female-containing (FC) families) and dashed (male-only (MO) families) lines. The number of families evaluated in each group, the relative risk (RR) to the child born first versus second after the second affected child, and the *P* value from a two-sided Fisher’s exact test are shown in the tables below each panel. Whiskers note the 95% confidence intervals around each recurrence rate estimate.

When comparing recurrence risk by birth order, between children born two versus one births after the second affected child, we find no significant differences in risk to females overall and to children in FC families. However, we observe that risk to males overall and risk to children in MO families shows a trend toward a decrease from the first to the second post-affected child (males: *P* = 0.042, adj. *P* = 0.33; MO: *P* = 0.028, adj. *P* = 0.23; see Figure 2A,B). When children are stratified by both sex and family type, we find that the risk to either males or females from FC families does not differ significantly between the first and second post-affected children. In contrast, risk to males in MO families drops for the second post-affected child, from 48.25% to 21.7% (*P* = 0.022, adj. *P* = 0.18; Figure 2C). These patterns are comparable when considering only the 83 families with at least two children born after their second affected child (Additional file 1: Figure S2A-C).

**Figure 3:**
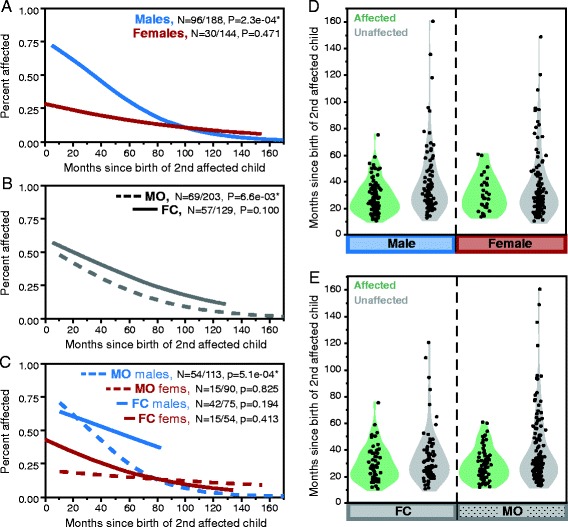
**Relationship between interbirth interval and ASD recurrence risk by sex and family type.** Recurrence risk to subsequent children decreases with increasing interval between births. **(A), (B)**, and **(C)** show the logistic regression estimated recurrence risk by the number of months since the birth of the second affected child within each subgroup of individuals as indicated by colors (blue for males, red for females) and solid (female-containing (FC) families) and dashed (male-only (MO) families) lines. *P* values from the whole-model test of logistic regression for affection status given the natural log of interbirth interval (months) are noted; fractions show the number of affected individuals out of the total within the indicated subgroup. **(D, E)** show the interbirth interval for all evaluated children given their affection status and either their sex **(D)** or family type **(E)**.

We also find a significant negative association between the number of months since the birth of the second affected child (IBI) and ASD risk to the next-born child (*X*^2^ = 10.41, *P* = 1.25e−03; Table 2). Within subgroups of children, this effect is significant for males overall (*P* = 2.31e−04; Figure 3A), for children in MO families (*P* = 6.63e−03; Figure 3B), and for male children in MO families (*P* = 5.1e−04; Figure 3C). The relationship between IBI and ASD status does not reach significance for any subgroup of females or children from FC families, consistent with the hypothesized existence of a maternal, uterine risk mechanism that predominantly affects MO families. However, when sex and family type (FC or MO) are both included as factors in the regression model for recurrence, only the main effects sex and IBI, not family type, are significant (Additional file 2: Table S3). We also note that the significance of these effects appears to be driven by unaffected children with long IBIs (Figure 3D,E); when we evaluate only those children born within 60 months of the second affected in their family, the associations between IBI and ASD risk diminish (males *P* = 0.05, females *P* = 0.600, FC *P* = 0.6, MO *P* = 0.4).

**Table 2:** **Autism recurrence risk by interbirth interval**

**Group**	***N***	**Chi-squared**	***P*** **value**	**Adjusted** ***P*** **value**
All	332	10.41	1.25e−03	0.011*
Males	188	13.56	2.31e−04	2.08e−03*
Females	144	0.52	0.471	1
FC	129	2.7	0.100	0.903
MO	203	7.37	6.63e−03	0.060
FC, Males	75	1.69	0.194	1
FC, Fems	54	0.67	0.413	1
MO, Males	113	12.08	5.10e−04	4.59e−03*
MO, Fems	90	0.049	0.825	1

Chi-squared statistics and *P* values are from the whole-model test for logistic regression of affection status of the first child born after the second affected child by the natural log of the interbirth interval (time since the birth of the second affected child) in months. Adjusted *P* values have been corrected for nine tests. *Adjusted *P* value ≤0.05. MO, male-only families; FC, female-containing families.

### Quantitative phenotypes

To determine if males and females or the different family types differed from one another in the presentation of ASD and its impact on functioning, we assessed quantitative measures of ASD severity, general functioning, and intellectual ability. To compensate for the non-independence of multiple children from each family, we compared VABS, PPVT, Raven NVIQ, and SRS scores from one randomly selected proband per family. We observed significantly higher VABS (better adaptive function) scores in probands from FC compared with MO families (average FC-MO difference = 4.42, *P* = 2.60e−03, adj. *P* = 0.042). This difference was also nominally significant when comparing only male probands (FC-MO difference = 2.85, *P* = 0.049). We also observed significantly lower VABS scores in males from FC families as compared with their sisters by a paired test (average difference = −2.85, *P* = 0.035). No comparison of scores from the PPVT, Raven’s NVIQ, and SRS showed any significant group differences on these measures.

### Concordance in twin pairs

**Figure 4:**
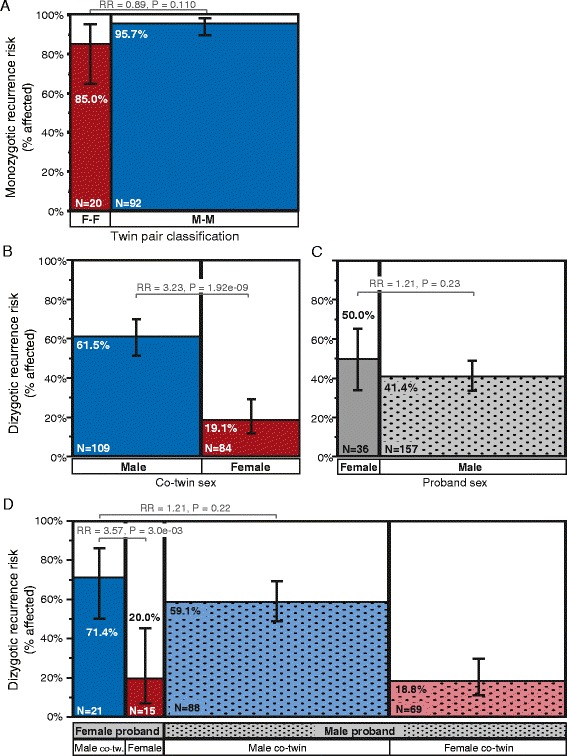
**Concordance rates in monozygotic and dizygotic twin pairs classified by their sex.** ASD concordance rates are higher for monozygotic than dizygotic twins, and concordance in dizygotic twins shows an effect of sex. **(A)** Mosaic plot shows the proportion of concordant monozygotic (MZ) twin pairs by the sex of the pair (F-F = female-female, M-M = male-male). Mosaic plots in **(B)**, **(C)**, and **(D)** show recurrence rates for ASD in co-twins from dizygotic (DZ) twin pairs by co-twin sex **(B)**, proband twin sex **(C)**, and by each combination of proband and co-twin sex **(D)**. Bar widths are proportional to the number of twin pairs in each group, which is also noted in the figure, and whiskers note the 95% confidence intervals around each recurrence risk estimate. Relative risk (RR) and *P* values for each comparison of interest are also noted in the figure.

We assessed concordance rates in 112 MZ multiples and 193 DZ twin pairs from AGRE. We identified high concordance rates in MZ multiples with male pairs (M-M) showing 95.6% concordance and female pairs (F-F) showing 85% concordance (F-F:M-M RR = 0.89, *P* = 0.11; Figure 4A; Additional file 2: Table S4A). For DZ twin pairs, we find a significantly higher recurrence rate among male co-twins than female co-twins (61.5% versus 19.1%; M:F RR = 3.23, *P* = 1.92e−09, adj. *P* = 7.66e−09; Figure 4B; Additional file 2: Table S4B); this sex difference is also apparent when co-twins of female probands are tested separately (71.4% versus 20.0%; M:F RR = 3.57, *P* = 2.97e−03, adj. *P* = 0.012; Figure 4D; Additional file 2: Table S4B). We also observe a trend toward higher recurrence rate in co-twins of female probands than male probands (50.0% versus 41.4%; F-pro:M-pro RR = 1.21, *P* = 0.23; Figure 4C; Additional file 2: Table S4B). Logistic regression for co-twin affection status corroborated these results by demonstrating only a significant main effect of co-twin sex (*P* = 3.45e−06); neither the proband sex nor the interaction (proband sex by co-twin sex) terms were significant in this model (Additional file 2: Table S4C).

## Discussion

The first main hypothesis derived from the female protective model is simply that males should demonstrate greater ASD risk than females. Our findings confirm this prediction, as we observe 2.25- to 2.6-fold increased recurrence risk to males compared with females. This increased risk for ASD in males compared with females corroborates findings from other studies of recurrence risk in infant siblings [6] as well as in a sample of families from AGRE and the Interactive Autism Network registry [5]. The observed recurrence rates of approximately 50% in males and 20% in females are also entirely consistent with those reported by Zhao *et al*. [28], whose earlier analysis also utilized a smaller sample of AGRE families available at the time. Additionally, recurrence rates in DZ twins confirm this hypothesis as well, with male co-twins showing a 3.23-fold increased risk compared with female co-twins.

The second primary hypothesis from the female protective model is that the siblings of female probands should demonstrate greater ASD risk than the siblings of male probands, which we observe. We note that only when all children born after the second affected child are included in the recurrence rate calculation does this comparison survive a conservative correction for multiple testing. In other family structures (last child from three-child families, last child from families of three or more, familial risk estimation), where only one child per family is permitted to contribute to the recurrence risk estimate, the differences between FC and MO families no longer reach significance. These shifts in statistical significance between family sets utilizing one or more later-born affected children in the recurrence risk estimate suggest that this analysis is likely to be underpowered. The findings from the analysis of 193 DZ twin pairs suggest a similar issue, as DZ twin pairs show a non-significant trend toward higher recurrence risk in co-twins of female than of male probands.

We also acknowledge that the maximum magnitude of the Carter effect that we are able to observe in these data is limited by our stratification approach, which considers the sex of only two, early-born probands per family, or one proband twin per twin pair. We apply this approach to avoid systematic increases in risk estimates for FC families and twins that result from using the same children to both stratify families and to calculate family-stratified recurrence rates. By the proband-based approach that we use, families that are found to carry female-penetrant risk loads *post hoc*, as evidenced by affected, later-born female children or affected female co-twins, may be grouped with families and twin pairs of exclusively male affected children for analysis. If these ‘misclassified’ families and twin pairs carry the sorts of high genetic risk loads that are responsible for the Carter effect, this will increase recurrence rate estimates among ‘MO’ families and attenuate the observable difference between FC and MO families and twins. Therefore, the observation of higher recurrence in imperfectly separated FC families or twins compared with MO that overcomes this counter-hypothetical skew introduced by our method can be interpreted as robust evidence of the Carter effect.

Our secondary hypotheses address the relationship between subject sex and familial genetic risk load. We predicted that females from FC families should show reduced risk compared with males from these same families, which is observed. Recurrence rates in later-born female children from FC families are significantly lower than for male children from these families and are also reduced in female co-twins of female probands, demonstrating the impact of female-protective mechanisms even within highly risk-loaded FC families and twins. The observation of a consistent direction of recurrence rate differences between FC and MO males and the differences that we observe when both sexes are considered suggest that the lack of a significant difference among males from different family types is likely to be a consequence of low power within this subset of samples. Analysis of multiplex families from other, larger collections will be necessary to conclusively reject the null for the impact of sex-differential familial liability on ASD risk in males.

Alternatively, the lack of a significant increase in risk to FC males over MO males may suggest a potential modification to the multiple threshold liability model. As opposed to conceptualizing genetic risk load severity as purely quantitative, there may be a locus-specific component such that females are only vulnerable to the effects of a subset of specific inherited risk variants that uniformly increase males’ risk. Risk variants may be assigned to one of the two classes: 1) variants that are penetrant in both males and females and 2) variants that are predominantly penetrant in males. These variant classes may be functionally delineated by the specific loci that harbor the risk variants, in that some loci increase ASD risk in both sexes (relative to sex-differential, baseline population risk) but females are nearly fully protected from variants at other loci. There are a handful of risk loci such as SHANK1 microdeletions [49] and 16p13.11 CNVs [50] that have been reported to follow such a pattern, where male carriers manifest ASD or other neurodevelopmental conditions and female carriers do not.

Of the four quantitative phenotypic measures of intellectual ability tested and for all comparisons of interest, only the VABS showed post-correction and nominally significant score differences. In agreement with our hypotheses, paired tests comparing VABS scores in male versus female siblings from FC families show nominally lower scores in brothers compared with their sisters, consistent with the prediction that males would be more severely impacted by female-penetrant risk loads. These data suggest that genetic and sex-differential risk loads in these families impact liability for the ASD phenotype, but that they have minimal to no consistent impact on the measureable degree of symptom severity, intellectual ability, or adaptive functioning.

Lastly, we posited that siblings of autistic males should show a stronger contribution from non-genetic, environmental risk factors on their liability for ASD than siblings of autistic females, whose risk is predicted to be more completely derived from a larger genetic liability. The finding that IBI is a significant predictor of ASD recurrence risk supports this hypothesis. In agreement with previous studies of population-based cohorts from California [40,42] and Norway [41], we observe a negative relationship between IBI and ASD risk, with no autistic cases born more than 75 months after their next eldest, autistic sibling and with children born after short IBIs showing the highest recurrence rates. We find that IBI is a significant predictor of affection status only for males, specifically those born into MO families. This finding is consistent with the idea of males as more vulnerable to risk factors in general, as well as with our hypothesis that lesser variant loads in MO families may leave room for contributions from non-genetic risk factors. Such non-genetic factors may include events in uterine or early postnatal development; maternal stress, inflammation, and deficiency of micronutrients such as folic acid have all been hypothesized as potential causes of the increased risk for ASD in children born after short IBIs [41,42,51]. Discordance patterns in DZ twins are also consistent with this concept of non-heritable risk factors from which females are protected, as female DZ twins are far less likely to be affected with ASD than their male co-twins. Currently, the roles of these proposed factors in ASD risk remain speculative. Future work is needed to definitively identify the potential maternal factors involved.

Previous studies report recurrence risks of 10% to 20% overall, far lower than observed here. These lower frequencies can most likely be attributed to the joint consideration of families with both inherited and *de novo* genetic risk architecture, as approximated by multiplex and simplex family structures, respectively. Supporting this, study of high-risk infant siblings reported greater recurrence risk in the subset of families with two or more elder affected of 32.2% overall and nearly 50% in males [6]; these rates are entirely consistent with what we observe here in AGRE.

Additionally, several studies of larger samples and population-based cohorts have tested and failed to observe a significant effect of older affected siblings’ sex on risk to later-born children [5,6,52,53]. One major difference between these study designs and that applied here is that we utilize two probands per family to classify families as FC or MO. By considering a greater number of affected children from each family, we are able to achieve a cleaner delineation between families with female-penetrant and male-specific risk loads. Also, the birth of an affected female child at any time in a family’s pedigree (or here within the first two affected, for methodological reasons) serves as a positive indicator of a high familial liability load that is likely to have a larger effect size as compared with the heritable variants carried by MO families on average. Therefore, genetic studies that focus on FC families may have increased power to detect and implicate heritable risk variants, which have so far remained largely elusive.

Previous work has reported closer to equal representation of autistic males and females among severely impacted cases with comorbid intellectual disability and a more pronounced male bias among high-functioning individuals [37,38]. We observe the opposite pattern, which may be characteristic of multiplex families or specific to AGRE. With regard to the reports from other samples on sex ratios in high- and low-functioning individuals, it has been suggested that current diagnostic tools are calibrated to a male-typical phenotype and that females are under-diagnosed for ASD due to their non-prototypical presentation of ASD symptoms [54,55]. For females who lack comorbid intellectual disability, diagnoses may be especially elusive. However, in this AGRE sample, the increased number of higher-functioning females may be a consequence of the ascertainment of families with multiple diagnosed children, and/or it may be that parents with an autistic child are more perceptive of symptoms in their daughters regardless of her intellectual ability.

The increased number of diagnosed females relative to males in this sample as compared with the general population (male/female relative risk of approximately 2.25 versus 4.5 in population samples from the United States [2]) may also be a consequence of an increased sensitivity to females’ symptoms. Findings from studies that are designed to evaluate all female and male subjects equivalently (as opposed to analyzing existing diagnostic records) support this possibility, including recent epidemiological population screens (South Korea, M:F = 2.5:1 [56]; Finland, M:F = 2:1 [57]) and a study of infant siblings of autistic probands (M:F = 1.65:1 [58]). It is also possible that male, latter-born children in multiple incidence families have less ASD risk, or that females have greater ASD risk, than their earlier-born siblings and that this change drives the attenuated relative risk that we observe in our sample. Here, we do observe a decline in male children’s recurrence rates between the first and second births after a second affected child (but no change in female children’s recurrence), consistent with such a sex-differential birth order effect on risk. Though here, this birth order effect was only a trend and thus warrants further investigation.

We also comment that the burden of care required by an autistic child can be substantial, and so, in addition to potential differences at the genetic risk level, there may be key differences between those parents who continue to have children after their earlier-born child is diagnosed with ASD and parents who do not. Though they may carry highly penetrant, heritable risk variants, the latter families will appear as simplex and are therefore not characterized here. A comparison of quantitative phenotypic measures in families who stopped versus continued having children after their second affected child does show trends of lower VABS scores in families who stopped, though these differences do not reach significance after adjustment for multiple testing (Additional file 2: Table S6).

## Conclusions

We characterize recurrence risk in a large cohort of uniformly ascertained multiplex families and observe significant sex differences in recurrence rates, with females showing a greater than twofold reduction in risk compared with males’ risk. We also observe higher recurrence rates in families with at least one affected female proband as compared with families whose probands are exclusively male; this difference is expected under the sex-differential threshold liability model for ASD. We further report a significant relationship between ASD and interbirth interval that is driven by male siblings of male probands. Taken together, these observations demonstrate that sex, genetic risk load, and putative environmental exposures all contribute to liability for ASD and that families with autistic females comprise a set of multiplex families enriched for larger genetic risk variant loads. Identification of female protective mechanisms in these high-risk families would open up new therapeutic windows, and the work to discover sex-differential heritable risk variant loads will advance our understanding of the shared familial component of ASD risk.

## **Additional files**

Additional file 1: Figures S1 and S2.
**Figure S1.** Filtering applied to select 1,120 multiplex families for analysis. **Figure S2.** Patterns of ASD risk to children born after the multiplex criterion are met in families with at least two subsequent births. Additional file 2: Tables S1 to S6.
**Table S1.** Recurrence risk in multiplex AGRE families by sex and family type. **Table S2.** Logistic regression for affection status by sex and family type. **Table S3.** Logistic regression for affection status by interbirth interval. **Table S4.** Concordance rates in monozygotic and dizygotic twin pairs. **Table S5.** Adaptive behavior, intellectual ability, and ASD symptoms by sex and family type. **Table S6.** Adaptive behavior, intellectual ability, and ASD symptoms by family stoppage status.

## Abbreviations

AGRE: Autism Genetics Resource Exchange
ASD: autism spectrum disorder
ATN: above the highest possible score for Raven’s Progressive Matrices normalized by age
BTN: below the lowest possible score for Raven’s Progressive Matrices normalized by age
CNV: copy number variant
DZ: dizygotic
FC: female-containing
IBI: interbirth interval
IPI: interpregnancy interval
IQ: intelligence quotient
M:F: male-to-female ratio
MO: male-only
MZ: monozygotic
NVIQ: non-verbal IQ
PDD-NOS: pervasive developmental disorder-not otherwise specified
PPVT: Peabody Picture Vocabulary Test
RR: relative risk
SNV: single nucleotide variant
SRS: Social Responsiveness Scale
VABS: Vineland Adaptive Behavior Scales
