# Supplementary material for: Recurrence rates provide evidence for sex-differential, familial genetic liability for autism spectrum disorders in multiplex families and twins
Source: Mol Autism. 2015 May 13;6:27. doi: 10.1186/s13229-015-0004-5 (PMC4429923; doi:10.1186/s13229-015-0004-5)
Supplement: Additional file 1: Figures S1 and S2. — Figure S1. Filtering applied to select 1,120 multiplex families for analysis. Figure S2. Patterns of ASD risk to children born after the multiplex criterion are met in families with at least two subsequent births. [file 13229_2015_4_MOESM1_ESM.pdf]

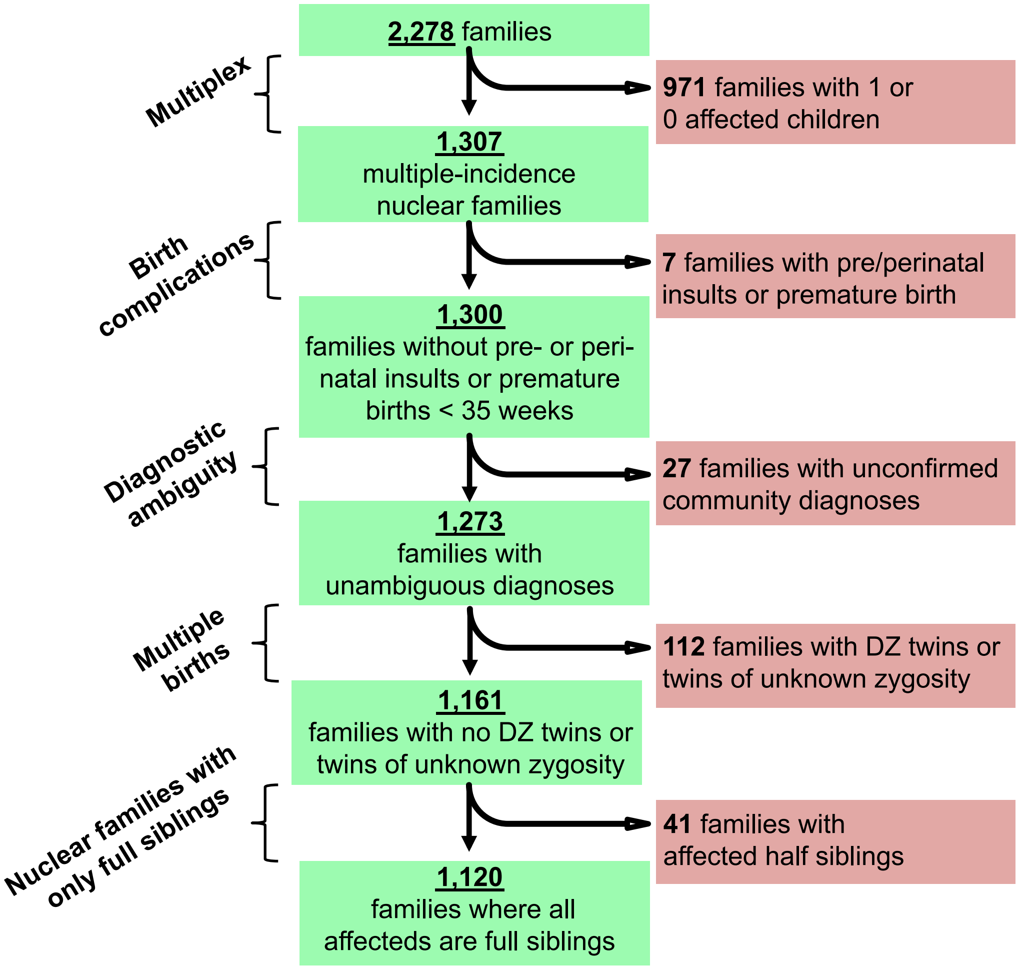

Figure S1: Filtering applied to select 1,120 multiplex families for analysis.
A series of exclusion criteria were applied to all 2,278 families enrolled in the AGRE collection to select a set of multiple-incidence nuclear families for evaluation of recurrence risk. The number of families passing each filter are shown in green, and the number of families filtered out at each step are shown on the right in pink.

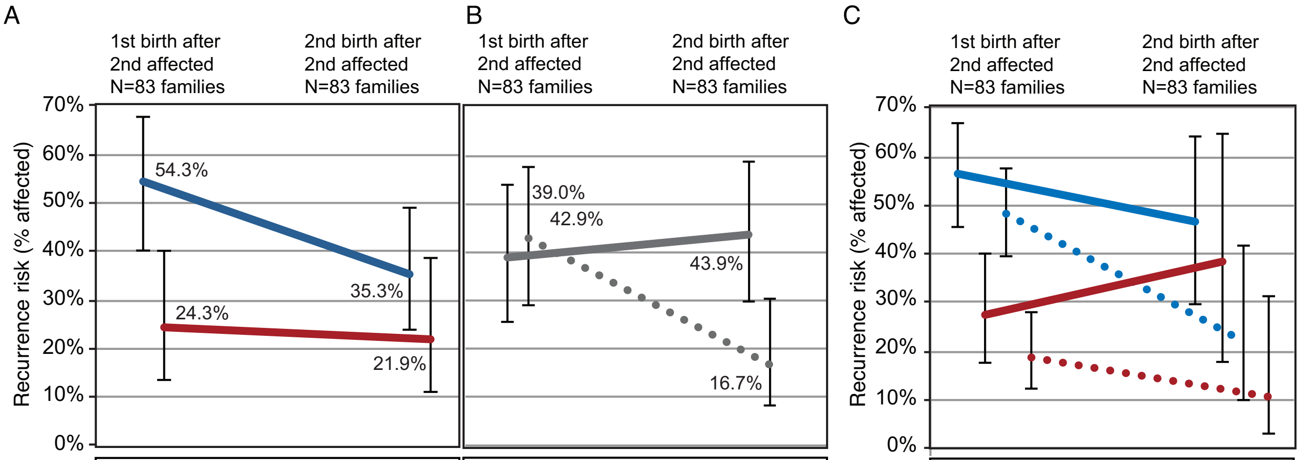

N:	1st	2nd	RR	P-val
Males	46	51	1.54	0.068
Fems	37	32	1.11	1.0

N:	1st	2nd	RR	P-val
MO	42	42	2.57	0.016
FC	41	41	0.89	0.82

N:	1st	2nd	RR	P-val
FC Males	24	28	1.08	1.0
FC Fems	17	13	0.61	1.0
MO Males	22	23	2.72	0.12
MO Fems	20	19	2.38	1.0

Figure S2: Patterns of ASD risk to children born after the multiplex criterion is met in families with at least two subsequent births.
After a family has 2 affected children, ASD recurrence risk is less for later-born males, particularly those in MO (male-only) families. Line graphs show the proportion of affected children born first and second after a second affected child in those families with at least two children born after their second affected child by A) sex, B) family type as FC (female-containing) or MO, and C) by sex within each family type. Line plots corresponding to each group are indicated by colors (blue for males, red for females) and solid (FC, female-containing families) and dashed (MO, male-only families) lines. The number of families evaluated in each group, the relative risk (RR) to the child born first versus second after the second affected child, and the p-value from a two-sided Fisher's exact test are shown in the tables below each panel. Whiskers note the 95% confidence intervals around each recurrence rate estimate.
