# Supplementary material for: Recurrence rates provide evidence for sex-differential, familial genetic liability for autism spectrum disorders in multiplex families and twins
Source: Mol Autism. 2015 May 13;6:27. doi: 10.1186/s13229-015-0004-5 (PMC4429923; doi:10.1186/s13229-015-0004-5)
Supplement: Additional file 2: Tables S1 to S6. — Table S1. Recurrence risk in multiplex AGRE families by sex and family type. Table S2. Logistic regression for affection status by sex and family type. Table S3. Logistic regression for affection status by interbirth interval. Table S4. Concordance rates in monozygotic and dizygotic twin pairs. Table S5. Adaptive behavior, intellectual ability, and ASD symptoms by sex and family type. Table S6. Adaptive behavior, intellectual ability, and ASD symptoms by family stoppage status. [file 13229_2015_4_MOESM2_ESM.pdf]

SUPPLEMENTAL TABLES

Table S1: Recurrence risk in multiplex AGRE families by sex and family type

A) All children born after 2nd affected. N=341 families, N=456 children, 36.0% affected

		N	Recur. rate	95% CI	Relative risk	P-value	Adj. P
**All**	**Males**	257	47.47%	41.45 – 53.57%	M/F		
	**Females**	199	21.11%	16.01 – 27.29%	2.25	3.11e-09	6.22e-08
**All**	**FC**	183	44.26%	37.26 – 51.50%	FC/MO		
	**MO**	273	30.40%	25.25 – 36.10%	1.46	1.78e-03	0.036
**Males**	**FC**	111	54.05%	44.80 – 63.03%	FC/MO		
	**MO**	146	42.47%	34.74 – 50.58%	1.27	0.043	0.86
**FC**	**Males**	111	54.05%	44.80 – 63.03%	M/F		
	**Females**	72	29.17%	19.94 – 40.51%	1.85	7.18e-04	0.014

B) First child born after 2nd affected. N=341 families, 38.4% affected

		N	Recur. rate	95% CI	Relative risk	P-value	Adj. P
**All**	**Males**	190	51.58%	44.51 – 58.58%	M/F		
	**Females**	151	21.85%	16.01 – 29.10%	2.36	1.21e-08	2.42e-07
**All**	**FC**	131	44.28%	36.05 – 52.82%	FC/MO		
	**MO**	210	34.76%	28.65 – 41.42%	1.27	0.051	1
**Males**	**FC**	76	56.58%	45.39 – 67.14%	FC/MO		
	**MO**	114	48.25%	39.28 – 57.32%	1.17	0.16	1
**FC**	**Males**	76	56.58%	45.39 – 67.14%	M/F		
	**Females**	55	27.27%	17.28 – 40.23%	2.07	7.19e-04	0.014

C) First child born after 2nd affected in families with exactly 3 children. N=198 families, 38.9% affected

		N	Recur. rate	95% CI	Relative risk	P-value	Adj. P
**All**	**Males**	111	52.25%	43.04 – 61.31%	M/F		
	**Females**	87	21.84%	14.45 – 31.61%	2.39	9.57e-06	1.91e-04
**All**	**FC**	72	48.61%	37.43 – 59.93%	FC/MO		
	**MO**	126	33.33%	25.70 – 41.95%	1.46	0.025	0.49
**Males**	**FC**	40	65.00%	49.51 – 77.87%	FC/MO		
	**MO**	71	45.07%	34.05 – 56.60%	1.44	0.034	0.68
**FC**	**Males**	40	65.00%	49.51 – 77.87%	M/F		
	**Females**	32	28.13%	15.56 – 45.37%	2.31	1.86e-03	0.037

D) First child born after 2nd affected is last child in family. N=258 families, 37.6% affected

		N	Recur. rate	95% CI	Relative risk	P-value	Adj. P
**All**	**Males**	144	50.69%	42.62 – 58.74%	M/F		
	**Females**	114	21.05%	14.58 – 29.42%	2.41	6.87e-07	1.37e-05
**All**	**FC**	90	46.67%	36.71 – 56.90%	FC/MO		
	**MO**	168	32.74%	26.10 – 40.15%	1.43	0.020	0.39
**Males**	**FC**	52	59.62%	46.07 – 71.84%	FC/MO		
	**MO**	92	45.65%	35.85 – 55.80%	1.31	0.075	1
**FC**	**Males**	52	59.62%	46.07 – 71.84%	M/F		
	**Females**	38	28.95%	17.00 – 44.76%	2.06	3.59e-03	0.072

E) Any 1 child selected after masking 2 affected children at random, 100 randomizations (Familial risk). N=556 families, 17.5% affected

		N	Recur. rate	95% CI	Relative risk	P-value	Adj. P
**All**	**Males**	270	25.56%	20.72 – 31.08%	M/F		
	**Females**	286	9.79%	6.86 – 13.79%	2.61	6.70e-07	1.34e-05
**All**	**FC**	208	22.60%	17.44 – 28.75%	FC/MO		
	**MO**	348	14.37%	11.07 – 18.44%	1.57	9.72e-03	0.19
**Males**	**FC**	99	31.31%	23.02 – 41.00%	FC/MO		
	**MO**	171	22.22%	16.64 – 29.03%	1.41	0.067	1
**FC**	**Males**	99	31.31%	23.02 – 41.00%	M/F		
	**Females**	109	14.68%	9.24 – 22.52%	2.13	3.38e-03	0.068

FC = female-containing family; MO = male-only family. Unadjusted P-values are from one-sided Fisher's exact tests for affection status by sex or by family type. Adjusted P-values have been corrected for 20 tests.

Table S2: Logistic regression for affection status by sex and family type

A) All children born after 2nd affected. N=341 families, N=456 children, 36.0% affected

Term	Estimate	Std Error	ChiSquare	Prob>ChiSq
Intercept	-0.662	0.109	37.16	1.09e-09
Sex [Male]	0.591	0.109	29.66	5.15e-08
Family type [FC]	0.299	0.109	7.61	5.80e-03
Sex[Male] by Family type [FC]	-0.066	0.109	0.37	0.54

B) First child born after 2nd affected. N=341 families, 38.4% affected

Term	Estimate	Std Error	ChiSquare	Prob>ChiSq
Intercept	-0.563	0.125	20.4	6.28e-06
Sex [Male]	0.660	0.125	28.06	1.18e-07
Family type [FC]	0.205	0.125	2.71	0.01
Sex[Male] by Family type [FC]	-0.038	0.125	0.09	0.76

C) First child born after 2nd affected in families with exactly 3 children. N=198 families, 38.9% affected

Term	Estimate	Std Error	ChiSquare	Prob>ChiSq
Intercept	-0.531	0.143	13.85	1.98e-04
Sex [Male]	0.720	0.143	25.44	4.56e-07
Family type [FC]	0.234	0.143	2.7	0.10
Sex[Male] by Family type [FC]	0.025	0.143	0.03	0.86

D) First child born after 2nd affected is last child in family. N=258 families, 37.6% affected

Term	Estimate	Std Error	ChiSquare	Prob>ChiSq
Intercept	-0.565	0.147	14.84	1.17e-04
Sex [Male]	0.673	0.147	21.03	4.52e-06
Family type [FC]	0.311	0.147	4.49	0.034
Sex[Male] by Family type [FC]	-0.029	0.147	0.04	0.84

E) Any 1 child selected after masking 2 affecteds at random, 100 randomizations (Familial risk). N=554 families, 17.5% affected

Term	Estimate	Std Error	ChiSquare	Prob>ChiSq
Intercept	-1.605	0.123	169.27	1.07e-38
Sex [Male]	0.586	0.123	22.55	2.05e-06
Family type [FC]	0.332	0.123	7.25	7.09e-03
Sex[Male] by Family type [FC]	-0.098	0.123	0.64	0.42

FC = female-containing family; MO = male-only family. Chi-squared statistics and unadjusted P-values are from the logistic regression for affection status by sex, family type, and the interaction between sex and family type.

Table S3: Logistic regression for affection status by inter-birth interval

Term	Estimate	Std Error	ChiSquare	Prob>ChiSq
**Intercept**	2.891	1.193	5.88	0.015
**ln(IBI)**	-0.657	0.278	5.6	0.018
**Sex [Male]**	0.699	0.131	28.56	9.08e-08
**ln(IBI) by sex [Male]**	-0.410	0.270	2.3	0.13
**Family type [FC]**	0.194	0.126	2.35	0.13
**Maternal age**	-0.029	0.036	0.68	0.41
**Paternal age**	-0.010	0.029	0.12	0.73

IBI = interbirth interval (months). FC = female-containing family, which is contrasted with families classified as male-only (MO). Estimates, chi-squared statistics, and P-values (Prob>ChiSq) are from a logistic regression model of affection status in the child born after the second affected child from each family (N=341) by the natural log of IBI in months, sex of the child, family type (FC or MO), maternal age, and paternal age at the time of the child's birth.

Table S4: Concordance rates in monozygotic and dizygotic twin pairs

A) Monozygotic twin pairs, N=112, 93.8% concordant

	N	Concordance	95% CI	Relative risk	P-value
Female-Female	20	85.00%	63.96 – 94.76%	F-F/M-M	
Male-Male	92	95.65%	89.35 – 98.30%	0.89	0.11

B) Dizygotic twin pairs, N=193 pairs, 43.0% concordant.

		N	Recur. rate	95% CI	Relative risk	P-value	Adj P
All	Male co-twin	109	61.47%	52.09 – 70.07%	M/F co-twin		
	Female co-twin	84	19.05%	12.08 – 28.72%	3.23	1.92e-09	7.66e-09
All	Co-twin of female proband	36	50.00%	34.47 – 65.53%	F/M proband		
	Co-twin of male proband	157	41.40%	33.99 – 49.22%	1.21	0.23	0.90
Male co-twin	Female proband	21	71.43%	50.04 – 86.19%	F/M proband		
	Male proband	88	59.09%	48.65 – 68.77%	1.21	0.22	0.86
Female proband	Male co-twin	21	71.43%	50.04 – 86.19%	M/F co-twin		
	Female co-twin	15	20.00%	7.05 – 45.19%	3.57	2.97e-03	0.012

C) Logistic regression for co-twin affection status by co-twin sex and proband sex in dizygotic twin pairs.

Term	Estimate	Std Error	ChiSquare	Prob>ChiSq
Intercept	-0.391	0.222	3.08	0.079
Co-twin sex [Male]	1.033	0.222	21.55	3.45e-06
Proband sex [Female]	0.156	0.222	0.49	0.48
Proband sex [Female] by co-twin sex [Male]	0.119	0.222	0.28	0.60

Unadjusted P-values in A) and B) are from one-sided Fisher's exact tests for co-twin affection status by co-twin sex or by proband twin sex. Adjusted P-values in B) have been corrected for 4 tests. In C), chi-squared statistics and P-values (Prob>Chisq) are from logistic regression for co-twin affection status by co-twin sex, proband twin sex, and the interaction between co-twin and proband sex.

Table S5: Adaptive behavior, intellectual ability, and ASD symptoms by sex and family type

A) Males vs. females, 1 randomly selected proband per family

	Males			Females			Males vs. females				
	N	Mean	SD	N	Mean	SD	Diff.	SE	t	P	Adj. P
VABS	587	60.91	18.62	177	64.34	20.92	-3.43	1.75	-1.96	0.051	0.82
PPVT	505	85.34	26.68	153	85.73	25.23	-0.39	2.36	-0.17	0.868	1
Raven's NVIQ	464	104.27	23.04	145	101.09	22.20	3.18	2.13	1.49	0.138	1
SRS	373	103.85	33.34	120	101.53	37.33	2.32	3.82	0.61	0.544	1

B) FC vs. MO, 1 randomly selected proband per family

	FC			MO			FC vs. MO				
	N	Mean	SD	N	Mean	SD	Diff	SE	t	P	Adj. P
VABS	293	64.47	20.40	471	59.99	18.25	4.42	1.46	3.02	2.60e-03	0.042
PPVT	255	87.24	24.72	403	84.29	27.27	3.13	2.06	1.52	0.130	1
Raven's NVIQ	245	103.03	21.20	364	103.83	23.94	-0.63	3.00	-0.34	0.733	1
SRS	193	99.53	36.13	300	105.70	32.95	-6.16	3.22	-1.91	0.057	0.91

C) FC vs. MO, 1 randomly selected male proband per family

	FC			MO			FC vs. MO				
	N	Mean	SD	N	Mean	SD	Diff	SE	t	P	Adj. P
VABS	255	62.79	18.79	472	59.86	18.16	2.85	1.45	1.97	0.049	0.79
PPVT	213	88.18	25.92	404	84.48	27.16	3.90	2.23	1.75	0.081	1
Raven's NVIQ	200	105.37	21.23	366	104.13	23.89	1.44	1.95	0.74	0.461	1
SRS	158	105.66	32.85	301	106.19	32.99	-0.53	3.23	-0.16	0.870	1

D) Males vs. females within FC families, 1 randomly selected affected brother & sister, paired test

	Males			Females			Males vs. females				
	N	Mean	SD	N	Mean	SD	Diff	SE	t	P	Adj. P
VABS	251	62.77	18.86	251	65.62	20.27	-2.85	1.34	-2.12	0.035	0.56
PPVT	171	89.91	25.04	171	89.89	24.49	0.02	2.28	0.01	0.992	1
Raven's NVIQ	163	107.20	20.99	163	103.53	23.39	3.67	2.22	1.65	0.100	1
SRS	142	107.27	33.01	142	104.93	35.33	2.34	3.82	0.61	0.542	1

FC, female-containing family; MO, male-only family; VABS, Vineland Adaptive Behavior Scales composite standard score; PPVT, Peabody Picture Vocabulary Test standard score; Raven NVIQ, Raven's Progressive Matrices estimated non-verbal intelligence quotient; SRS, Social Responsiveness Scale raw total score. T statistics and unadjusted P-values are from two-sided t-tests allowing for unequal variances for affection status by sex or by family type. For D), a paired t-test was used to compare affected males and females from the same families. Adjusted P-values have been corrected for 16 tests.

Table S6: Adaptive behavior, intellectual ability, and ASD symptoms by family stoppage status

A) Families who stopped vs. continued having children, 1 randomly selected proband per family

	Stop			Continue			Stop vs. continue				
	N	Mean	SD	N	Mean	SD	Diff.	SE	t	P	Adj. P
**VABS**	533	60.9	19.0	231	63.6	19.6	-2.71	1.53	-1.77	0.077	0.82
**PPVT**	461	84.4	26.9	197	87.9	24.9	-3.56	2.17	-1.64	0.102	1
**Raven NVIQ**	421	103.2	23.6	188	104.1	21.1	-0.86	1.92	-0.45	0.653	1
**SRS**	353	101.5	33.6	140	107.9	35.8	-6.45	3.51	-1.83	0.068	1

B) Families with ≥3 full sibling children who stopped vs. continued having children, 1 randomly selected proband per family

	Stop			Continue			Stop vs. continue				
	N	Mean	SD	N	Mean	SD	Diff.	SE	t	P-value	Adj. P
**VABS**	138	57.8	20.2	231	63.6	19.6	-5.75	2.15	-2.68	7.90e-03	0.13
**PPVT**	114	82.4	30.3	197	87.9	24.9	-5.55	3.35	-1.66	0.099	1
**Raven NVIQ**	112	101.5	27.2	188	104.1	21.1	-2.63	2.99	-0.88	0.380	1
**SRS**	92	103.7	34.7	140	107.9	35.8	-4.23	4.72	-0.90	0.371	1

C) FC Families with ≥3 full sibling children who stopped vs. continued having children, 1 randomly selected proband per family

	Stop			Continue			Stop vs. continue				
	N	Mean	SD	N	Mean	SD	Diff.	SE	t	P	Adj. P
**VABS**	43	56.2	22.8	99	67.0	20.2	-10.73	4.03	-2.66	9.60e-03	0.15
**PPVT**	35	86.1	28.1	87	89.8	23.8	-4.03	5.40	-0.75	0.459	1
**Raven NVIQ**	38	101.3	23.9	84	103.9	21.7	-2.88	4.55	-0.63	0.529	1
**SRS**	32	109.2	29.7	51	101.3	38.3	7.85	7.51	1.05	0.299	1

D) MO Families with ≥3 full sibling children who stopped vs. continued having children, 1 randomly selected proband per family

	Stop			Continue			Stop vs. continue				
	N	Mean	SD	N	Mean	SD	Diff.	SE	t	P	Adj. P
**VABS**	95	58.6	19.0	132	61.0	18.7	-2.54	2.54	-1.00	0.318	1
**PPVT**	79	80.7	31.2	110	86.5	25.8	-5.50	4.28	-1.28	0.201	1
**Raven NVIQ**	74	101.6	28.9	104	104.3	20.7	-2.49	3.92	-0.64	0.527	1
**SRS**	60	100.7	37.0	89	111.7	33.9	-10.93	5.98	-1.83	0.070	1

VABS = Vineland Adaptive Behavior Scales composite standard score; PPVT = Peabody Picture Vocabulary Test standard score; Raven NVIQ = Raven's Progressive Matrices estimated non-verbal intelligence quotient; SRS = Social Responsiveness Scale raw total score. T statistics and unadjusted P-values are from two-sided t-tests allowing for unequal variances for family's stoppage status. Adjusted P-values are corrected for 16 tests.
